# Modeling public trust in AI cognitive capabilities using statistical and machine learning approaches

**DOI:** 10.1038/s41598-025-23447-4

**Published:** 2025-11-13

**Authors:** Reshaa F. Alruwaili, Abdullah A. Alasmari, Hussien Tash Niyazi, I. K. Youssef, Hanan Aifan, Somia A. Asklany

**Affiliations:** 1 Department of Psychology, College of Social Sciences, Imam Mohammad Ibn Saud Islamic University (IMSIU), Riyadh, Saudi Arabia; 2 Department of Mathematics, Faculty of Science, Islamic University of Madinah, Madinah, Saudi Arabia; 3 Department of Curriculum and Teaching, College of Education, Najran University, Najran, Saudi Arabia; 4 Department of Computer Science, College of Science, Northern Border University, Arar, Saudi Arabia

**Keywords:** Artificial intelligence (AI), Cognitive capabilities, Statistical analysis, Machine learning, Human–AI interaction, Human behaviour, Statistics

## Abstract

As artificial intelligence (AI) systems increasingly perform cognitive functions, assessing public trust in these capabilities is critical. This study investigates the impact of age, gender, and familiarity with AI on confidence in AI’s ability to make simple decisions, complex judgments, and perform memory recall tasks. A survey of 400 participants was analyzed using statistical tests and a Random Forest classifier. Results indicate that AI familiarity is the strongest predictor of confidence, followed by age and gender. Participants expressed greater trust in AI for factual, memory-based tasks, and preferred human decision-making in high-stakes scenarios such as medical diagnosis and autonomous driving. The Random Forest model demonstrated strong predictive performance, confirming that familiarity and age are the most influential predictors of trust. These findings highlight the nuanced role of demographic and experiential factors in shaping trust in AI’s cognitive capabilities and provide practical implications for designing user-aligned, trustworthy AI systems.

## Introduction

Artificial intelligence (AI) systems now underpin critical decision-making across many healthcare, finance, and transportation domains. While AI offers enhanced efficiency and accuracy, its adoption hinges on user trust, which can be influenced by factors including prior exposure to AI, individual demographics, and task characteristics^1–3^. Prior work demonstrates that repeated interactions with AI tools increase reliance^4^ and that age and education level moderate trust behaviors. Yet these studies typically analyze predictors separately, leaving the combined effects on trust in different cognitive domains underexplored.

Recent syntheses highlight that trust in AI remains a dynamic, multi-dimensional construct shaped by both technical and social factors^5^. This work underscores the need for integrative approaches that combine psychological, organizational, and computational perspectives.

This study bridges that gap by employing a multimethod framework: we first conduct a multivariate analysis of variance (MANOVA) to assess how demographic factors (age, gender, education), AI familiarity, and task complexity jointly influence trust in AI-assisted tasks, including simple decisions, complex judgments, and memory recall. We then apply Random Forest classification to predict high versus low trust profiles based on the same variables, evaluating model performance via cross-validation. This integrated approach enables both statistical inference and predictive insight into trust dynamics.

We address three core questions: (1) What is the relative impact of demographic and experiential factors on trust in AI? (2) Does task complexity moderate these relationships across cognitive tasks? (3) How accurately can machine learning classifiers distinguish trust levels based on user characteristics? By synthesizing rigorous hypothesis testing with modern predictive analytics, our findings aim to guide the development of human-centered AI systems that calibrate trust appropriately across diverse user populations and application contexts.

## Literature review

A robust body of research has examined determinants of trust in AI and automated systems. Early work conceptualized trust calibration in automation, emphasizing that appropriate reliance requires accurate mental models of system capabilities^6^. Building on this foundation, quantitative work on explainability argues that transparency interventions can mitigate user skepticism. More recent studies have highlighted demographic moderators: older adults often exhibiting lower initial trust but greater sensitivity to system feedback^7^, and educational attainment correlating positively with perceived algorithmic competence^8^.

More recently, transparency has been conceptualized not as a one-off disclosure but as an ongoing pipeline for building trust, embedded into iterative, contextualized interactions^9^.

Task context also plays a pivotal role^10^, demonstrating that users’ algorithm aversion decreases when they can adjust model outputs, suggesting control mechanisms enhance acceptance in decision-support scenarios. extended this by showing that trust varies across cognitive domains: users are more willing to delegate repetitive, low-stakes tasks than complex, high-stakes judgments. Complementing these experimental findings, several field studies in healthcare settings report that clinicians’ trust in diagnostic AI aligns with perceived outcome transparency and error tolerance levels^11^.

Despite these advances, few investigations have simultaneously modeled demographic, experiential, and task-related variables within a unified analytical framework. Our study addresses this gap by integrating multivariate hypothesis testing with predictive modeling to capture interactive effects on trust across diverse task types.

Cheng et al.^12^ proposed a framework for quantifying trustworthiness in neural networks, highlighting the role of opinion dynamics and uncertainty modeling in trust calibration. Shin^13^ explored how misinformation and human–algorithm interaction shape trust online, while Shin^14^ examined how innovation and sustainability intersect with AI bias and public trust.

In addition to these model-centric approaches, evidence suggests that miscalibration of AI confidence itself can distort collaboration outcomes. Both overconfident and underconfident systems reduce task effectiveness by encouraging either excessive reliance or unwarranted skepticism^15^.

Beyond individual and model-centric determinants, public attitudes and governance considerations also shape trust in AI. Large-scale survey evidence indicates that perceptions of risk and support for regulation co-evolve with trust in “digital minds,” underscoring the need to situate user-level findings within broader societal contexts^16^.

In this study, we follow human–automation research in defining trust as a user’s willingness to rely on an AI system under uncertainty, shaped by perceived competence, predictability, and value alignment. Because trust is partly subjective, its measurement typically relies on self-report scales complemented by behavioral or outcome-based indicators. Recent work also investigates quantifying trust and trustworthiness in models by explicitly modeling opinion uncertainty and confidence (e.g., Bayesian and evidential approaches), offering complementary perspectives to survey-based measures. Our operationalization multi-item Likert ratings of confidence in AI across cognitive tasks, analyzed with inferential statistics and predictive modeling, aligns with this literature while focusing on user-perceived capability. We contrast our approach with model-centric trust quantification frameworks and situate it within broader debates on misinformation, governance, and debiasing in human–computer interaction.

## Methodology

### Survey design

This study employed a structured online survey to assess public trust in AI’s cognitive capabilities. The instrument was designed to measure confidence in AI across three domains: simple decision-making, complex decision-making, and memory recall. The survey consisted of four sections:

1. Demographics and AI familiarity: Collected participants’ age, gender, educational background, and self-reported familiarity with AI on a 5-point Likert scale.
2. AI usage behavior: Measured frequency and context (personal, professional, or both) of AI technology use.
3. Trust in AI cognitive tasks: Assessed confidence in AI’s ability to perform simple decisions, complex decisions, and memory tasks using a 6-point Likert scale (1 = Not Confident to 6 = Extremely Confident).
4. Scenario-based comparisons: Participants indicated whether they trusted AI more, humans, or both equally across five real-world scenarios (e.g., self-driving, medical diagnosis).

The survey was piloted with 15 individuals to ensure clarity and validity, then distributed online through academic and social channels. A total of 400 valid responses were collected.

### Participants

In this study, a total of 400 adults aged 18 years and older were recruited via convenience sampling. Participants represented diverse backgrounds in terms of age, gender, education level, and prior exposure to artificial intelligence. Inclusion criteria required fluency in English and an age of at least 18 years. All participation was voluntary, and respondents provided informed consent through the approval granted by the Deanship of Scientific Research, Standing Committee for Scientific Ethics (Approval No. 1668; March 14, 2025).

### Data processing

Data was collected using Google Forms and processed with Python libraries including pandas, scipy, and statsmodels. Responses were cleaned, encoded numerically, and validated. Ambiguous answers were excluded. Familiarity levels and trust scores were treated as ordinal variables.

### Statistical analysis

Three main statistical methods were employed:

- MANOVA: Tested the multivariate impact of age, gender, and AI familiarity on confidence scores across the three task types.
- Kruskal–Wallis H-Tests: Assessed confidence score differences across age brackets for each task.
- Mann–Whitney U Tests: Compared trust scores between binary groups (e.g., male vs. female; high vs. low familiarity), with Bonferroni correction applied for multiple comparisons.

### Machine learning modeling

**Fig. 1:**
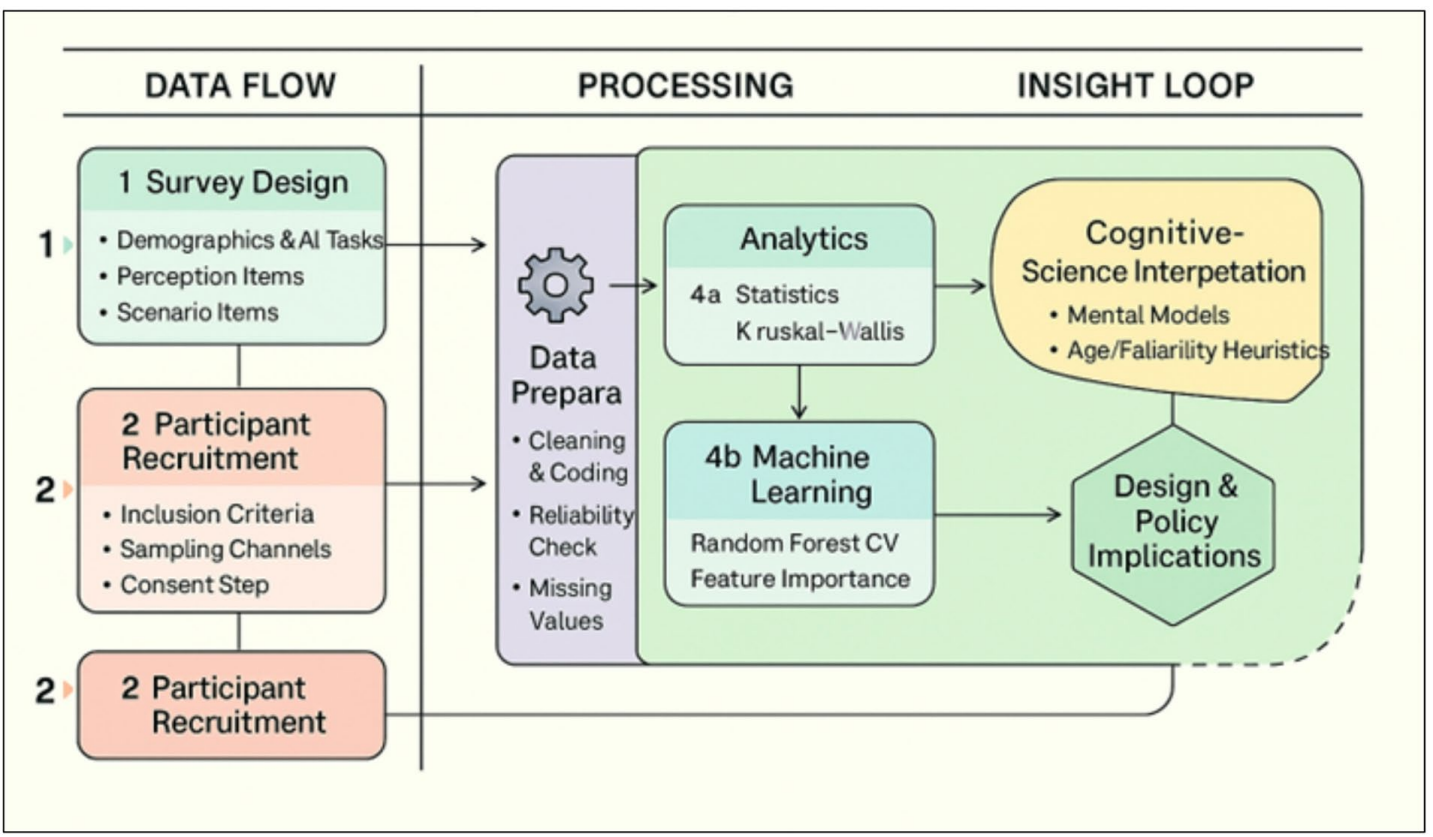
Flowchart of the proposed study design and analysis process.

Random Forest Classifier was trained to predict binary trust levels (high vs. low) to complement statistical inference. Features included demographics and AI usage behavior. Model performance was evaluated using precision, recall, F1-score, and feature importance. The flow chart of the proposed work is shown in Fig. 1. To assess robustness, we used stratified five-fold cross-validation with folds preserving the proportion of the binary trust label. Feature preprocessing was fitted only on the training folds and applied to the corresponding held-out fold to prevent leakage. We tuned hyperparameters (number of trees, max depth, min samples per split) via grid search nested inside the cross-validation. Class imbalance was addressed by (i) reporting per-class metrics and (ii) using class-balanced sample weights during training. Alongside accuracy, we report macro-averaged F1, AUROC, and area under the precision–recall curve (AUPRC) with 95% CIs from 1000 bootstrap resamples of the out-of-fold predictions. Finally, we examined calibration (Brier score and Expected Calibration Error) and presented reliability curves. These procedures reduce the risk that high accuracy is due to thresholding, imbalance, or inadvertent information leakage.

### Ethical considerations

All experimental procedures were performed strictly according to the relevant institutional and national guidelines and regulations. Ethical approval was obtained from the Institutional Review Board at Imam Mohammad ibn Saud Islamic University (Approval No. 1668).

## Results

### Descriptive statistics

The final sample included 400 participants, with a near-balanced gender distribution and wide age representation (18–55+). Most respondents reported moderate familiarity with AI and indicated using AI tools for personal and professional purposes.

### Trust in AI vs. human scenarios

Participants were asked to compare their trust in AI versus human decision-makers across five scenarios: self-driving, medical diagnosis, historical recall, logistics planning, and personal memory recall.

**Fig. 2:**
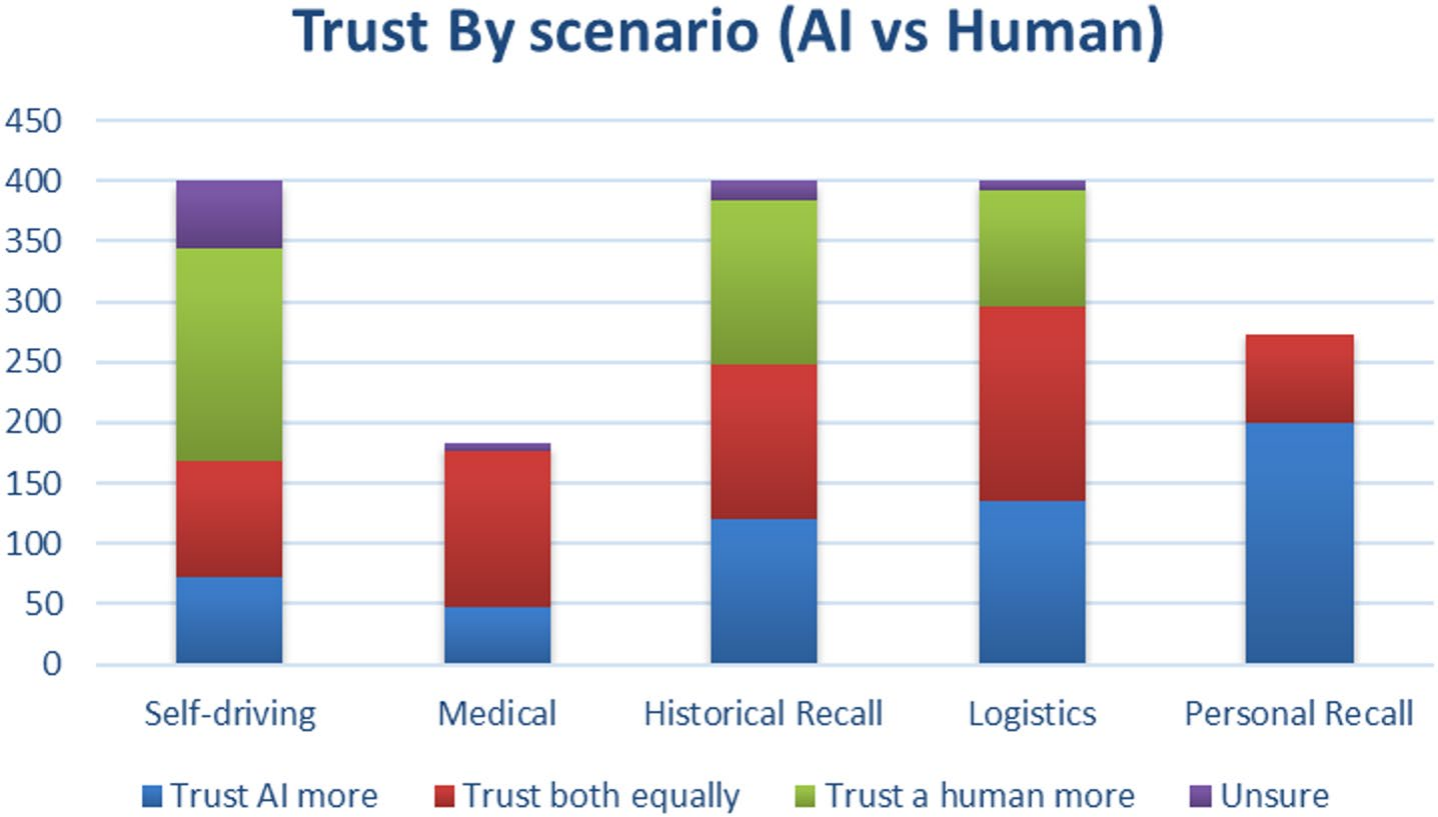
Trust in AI vs. human scenarios.

A stacked bar chart (Fig. 2) revealed that:

- Trust in humans was higher in medical and self-driving contexts.
- Trust in AI was more pronounced in memory-based scenarios (historical and personal recall).
- Many participants expressed equal trust in AI and humans in logistics tasks.

From a cognitive-science perspective, this study can be framed as three interrelated tasks:

1. Assessment of declarative memory recall.

**Fig. 3:**
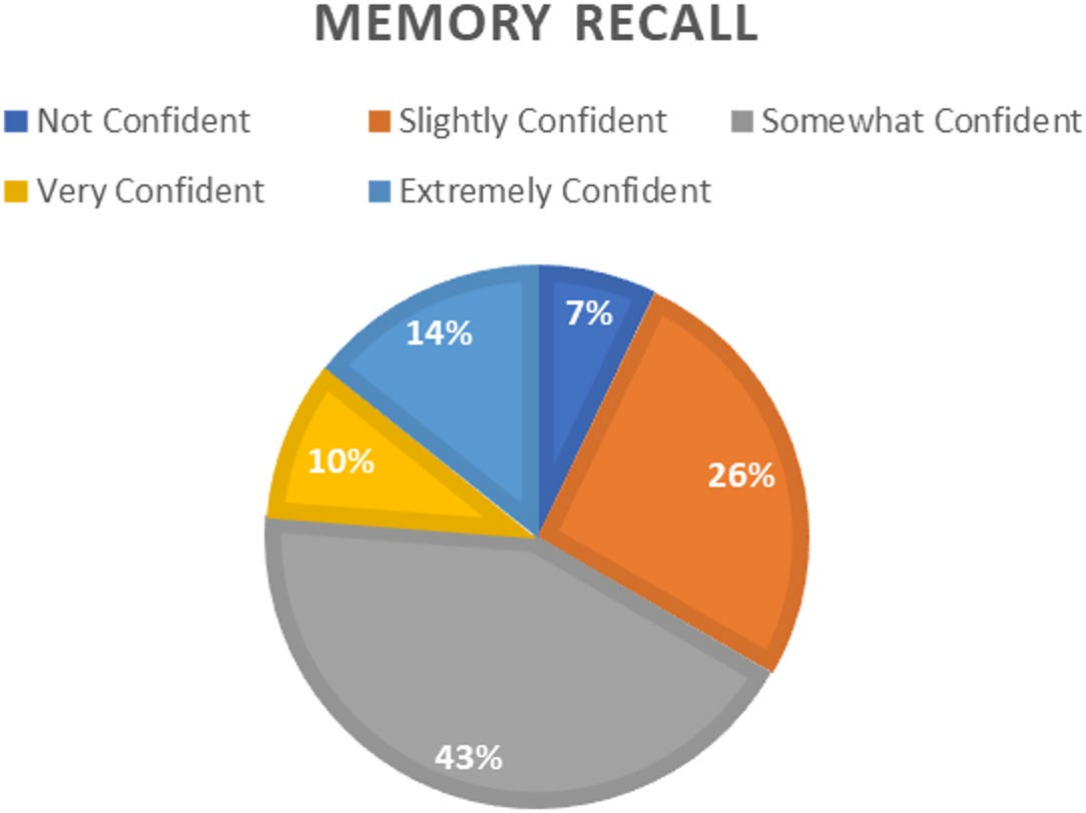
Memory recall distribution.

Examine how individuals gauge AI’s capacity to retrieve and present factual information analogous to human episodic and semantic memory processes. By analyzing confidence ratings and distribution as in Fig. 3, we probe whether AI’s memory-like functions align with cognitive models of recall accuracy and information retrieval fluency.

#### Evaluation of routine decision-making

**Fig. 4:**
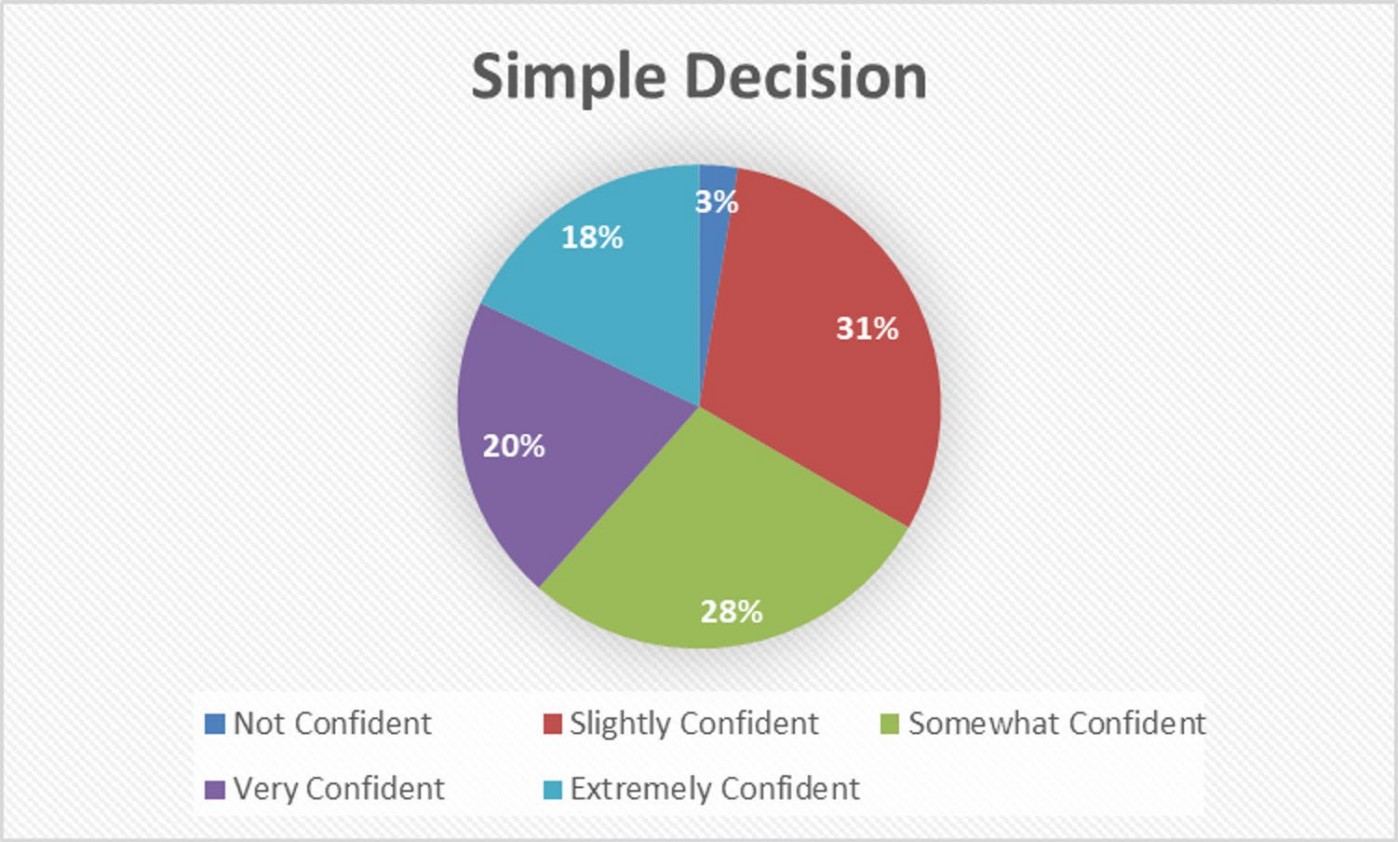
Simple decision distribution.

Investigate participants’ trust in AI for low-stakes, heuristic‐driven choices (e.g., product recommendations), which mirror System 1 processing in dual‐process theory. The Simple Decision distribution (Fig. 4) reveals how cognitive load and prior experience shape reliance on automatic AI suggestions versus intuitive human judgments.

**Fig. 5:**
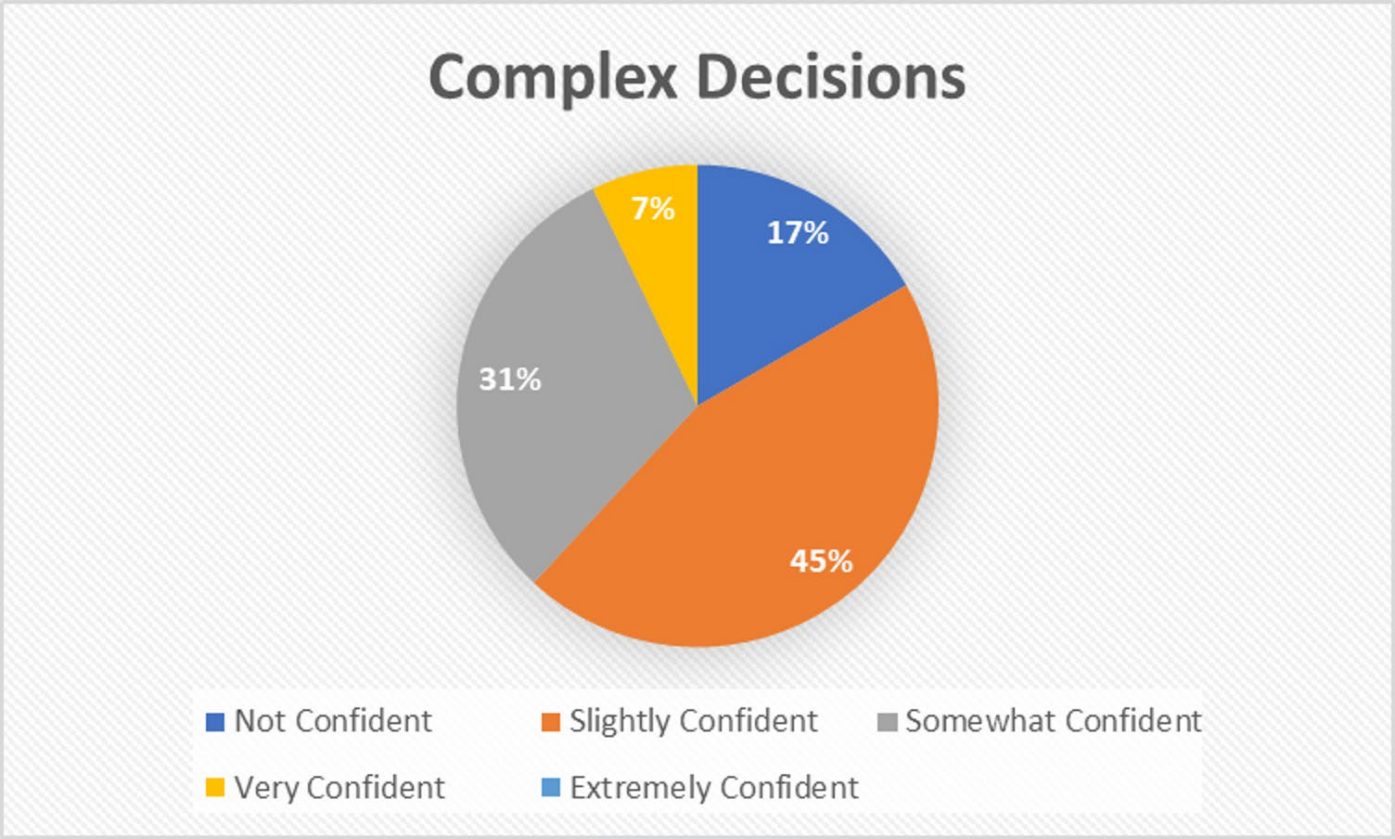
Complex decisions distribution.

1. Judgment of complex decisions.
2. Explore public skepticism toward AI’s capacity for nuanced, context-sensitive reasoning akin to System 2 cognition tasks requiring deliberation, ethical appraisal, and error monitoring. The Complex Decisions distribution, Fig. 5, captures the cognitive bias and risk‐aversion factors influencing whether individuals delegate responsibility to algorithmic agents in scenarios demanding deep expertise.

## Familiarity distribution

**Fig. 6:**
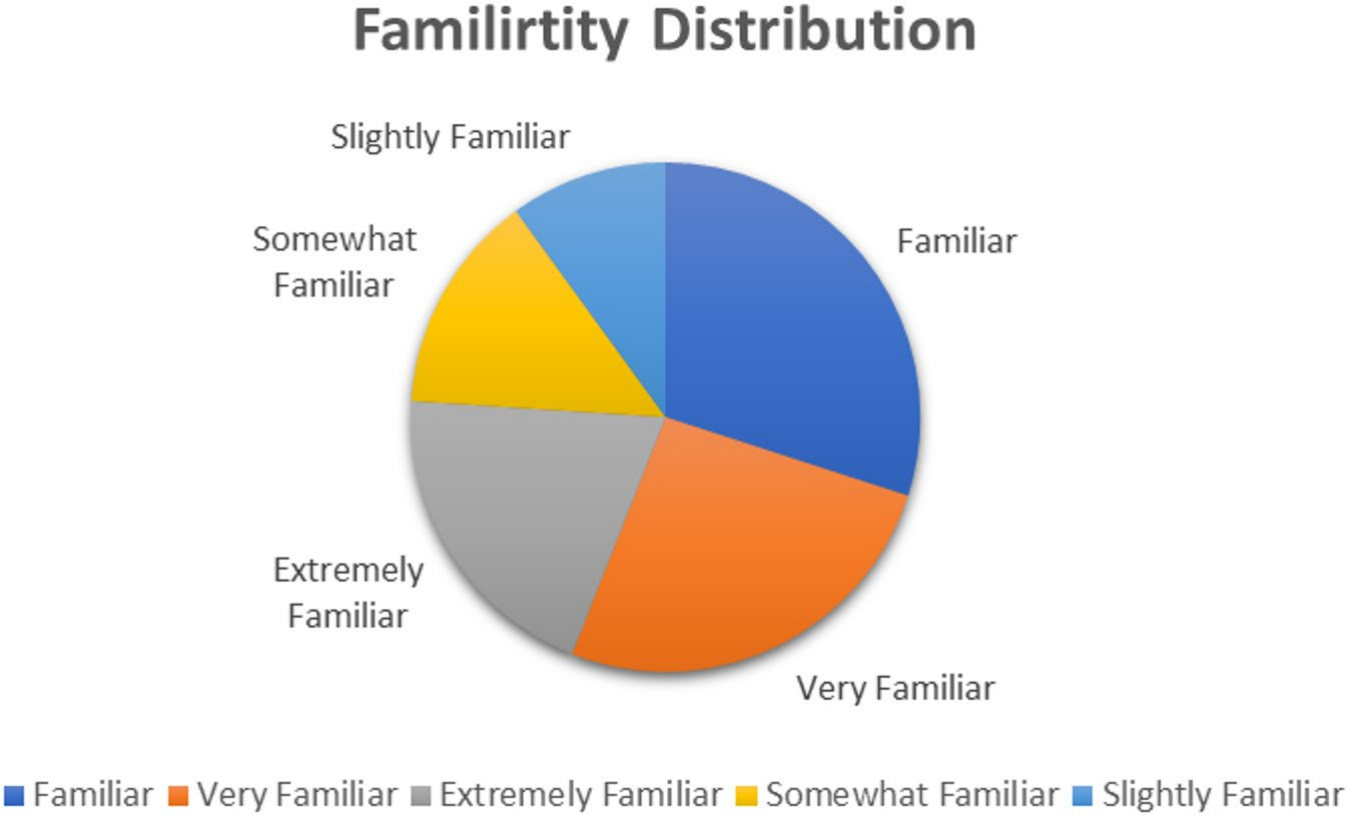
Familiarity distribution.

Explore the spectrum of prior AI exposure among participants, reflecting their self‑assessed knowledge and hands‑on experience with algorithmic systems. The Familiarity Distribution (Fig. 6) captures how varying levels of AI encounter, from minimal awareness to extensive “power user” engagement, shape individuals’ baseline readiness to trust machine‑driven recommendations. A peak in the mid‑range indicates a normative cohort with moderate AI literacy. At the same time, the pronounced upper tail highlights a subset of highly familiar users whose deep interaction history may predispose them to greater reliance on simple and complex AI tasks. Conversely, the lower end of the scale represents novices whose limited exposure likely contributes to heightened skepticism and algorithm aversion. By charting these familiarity profiles, Fig. 6 provides critical insight into the experiential dimension of trust formation and guides our predictive modeling of high-versus low‑trust user segments based on AI familiarity.

### Statistical analysis of confidence scores

To assess demographic influences on trust, we applied a combination of inferential tests:

#### Kruskal–Wallis H-Test

We tested whether confidence in AI differed significantly across age groups. Table 1 shows that the results were highly significant.

**Table 1:** Kruskal–Wallis H-Test results.

Task type	Test statistic	*p*-value	Result
Simple decision-making	56.82	1.35 × 10^−11^	Highly significant
Complex decision-making	23.86	8.54 × 10^−5^	Highly significant
Memory recall	30.34	4.18 × 10^−6^	Highly significant

**Fig. 7:**
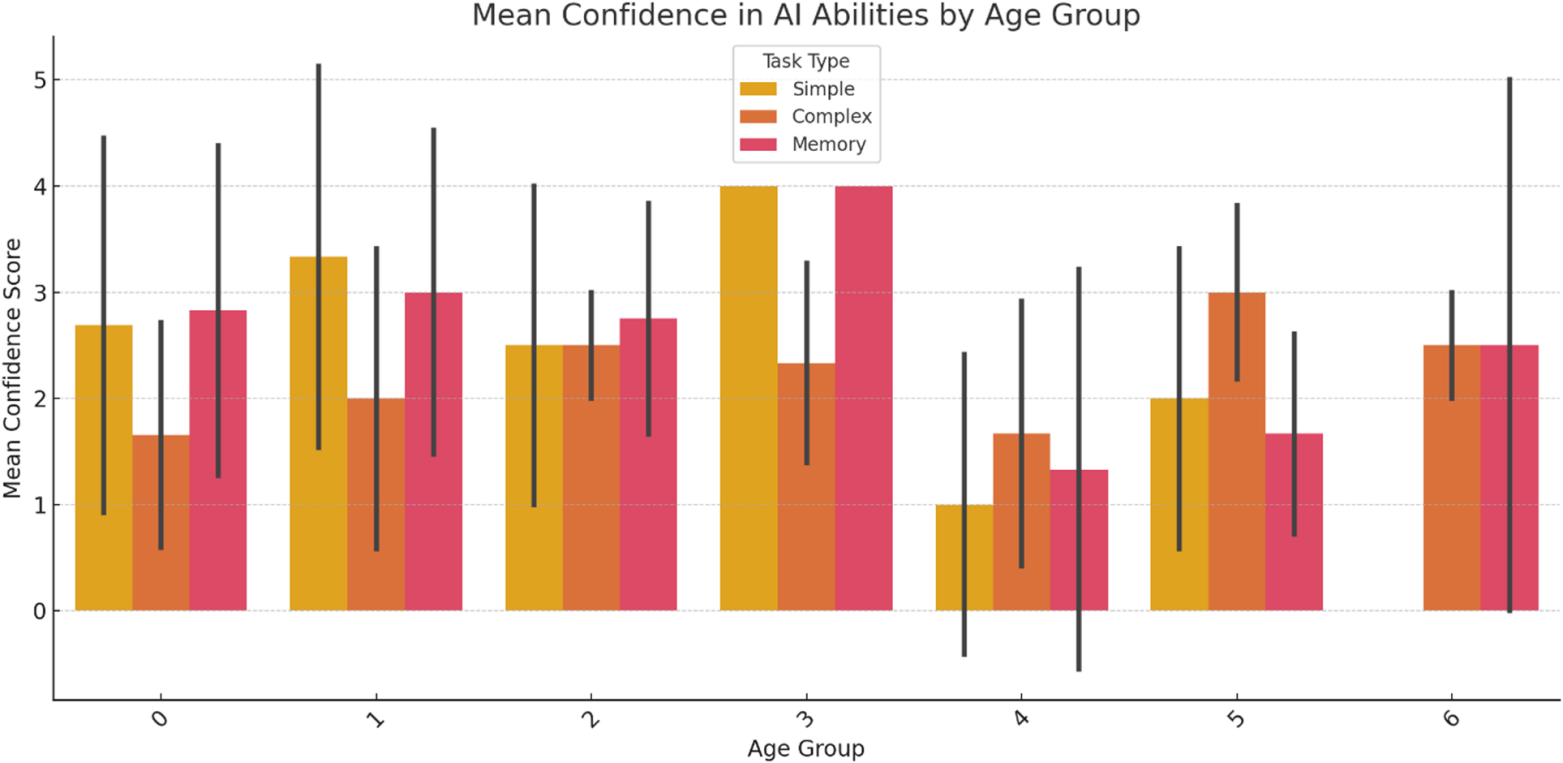
Mean confidence in AI by age group.

These results confirm that trust in AI varies notably by age, especially in simple decisions, as shown in Fig. 7.

#### MANOVA

A multivariate analysis of variance examined how age, gender, and familiarity with AI jointly influenced confidence in AI. Table 2 summarizes the results.

**Table 2:** Three variables confidence in AI.

Factor	Wilks’ Lambda	Significance (*p*)
Age	0.925	0.001
Gender	0.983	0.047
AI familiarity	0.806	0.001

AI familiarity had the strongest multivariate effect, followed by age and gender.

#### Mann–Whitney U test results with bonferroni correction

To compare confidence in AI’s abilities across different demographic groups (gender, age, and familiarity), Mann–Whitney U tests were conducted for each task. Bonferroni correction was applied to adjust for multiple comparisons. The results are summarized in Table 3.

**Table 3:** Mann–Whitney U test.

Task type	Group compared	U statistic	Raw *p*-value	Adjusted *p*-value	Significance
Simple decisions	Gender	6240.0	0.0064	0.0192	Statistically significant
Complex decisions	Gender	11712.0	0.2743	0.8230	Not significant
Memory recall	Gender	9888.0	0.6250	1.0000	Not significant
Simple decisions	Age	9952.0	0.3180	0.9540	Not significant
Complex decisions	Age	8704.0	< 0.0001	< 0.0001	Highly significant
Memory recall	Age	8992.0	0.00092	0.00276	Statistically significant
Simple decisions	Familiarity	15808.0	< 0.0001	< 0.0001	Highly significant
Complex decisions	Familiarity	16768.0	0.00077	0.00232	Statistically significant
Memory recall	Familiarity	18816.0	< 0.0001	< 0.0001	Highly significant

### Predictive modeling with random forest

We implemented a Random Forest Classifier using demographic and behavioral data to predict high vs. low trust in AI. Table 4 summarizes key findings.

**Table 4:** Classification performance.

Class	Precision	Recall	F1-Score	Support
Trust human/equal (0)	1.00	0.98	0.99	96
Trust AI more (1)	0.92	1.00	0.96	24
Accuracy			98.3%	120

Table 5 gives the top predictive features (importance).

**Table 5:** Top predictive features.

Feature	Importance
AI familiarity	32.4%
Age	30.0%
Context of AI use	13.7%
Gender	13.0%
Frequency of AI use	10.9%

Interpretation:

- The model performs very accurately (F1 = 0.98) in predicting trust in self-driving AI.
- Familiarity with AI and age are the most influential predictors, consistent with statistical results.
- Other demographic factors (context, gender, usage frequency) also contribute moderately.

## Discussion

This study used statistical and machine learning analyses to investigate public trust in AI’s cognitive capabilities across various domains. The results offer a comprehensive understanding of how age, gender, and familiarity with AI influence confidence in AI systems.

### Influence of demographics on trust

Our results showed that AI familiarity is the strongest predictor of confidence, a finding confirmed by MANOVA and the Random Forest classifier. Participants who reported higher familiarity with AI consistently expressed greater trust across all task types, especially in memory recall and logistics tasks, where AI is perceived to excel.

Age also emerged as a significant factor: younger participants showed higher confidence in AI’s decision-making abilities, particularly in simple and memory-based tasks. This trend aligns with previous studies suggesting that younger demographics are more comfortable with digital technologies and automation.

Although gender effects were smaller than those of familiarity and age, they were statistically detectable in multivariate tests. Prior work has linked gender differences to perceived risk, domain familiarity, and socialization surrounding automation. Future studies should investigate whether design affordances (e.g., error transparency, controllability) mitigate these gaps and whether their effects persist after establishing measurement invariance across gender.

### Trust by scenario type

Participants were more inclined to trust humans in high-stakes contexts such as medical diagnosis and self-driving decisions, echoing existing literature on algorithm aversion and the perceived need for human empathy, accountability, and nuance in critical scenarios.

In contrast, trust in AI exceeded human trust in domains requiring data recall and consistency, such as historical knowledge and memory retrieval. These results confirm the hypothesis that people trust AI more in tasks perceived as objective, factual, and repeatable.

### Predictive modeling insights

The Random Forest model demonstrated strong accuracy in classifying trust levels and identified AI familiarity, usage frequency, and age as top predictors. This supports the idea that trust in AI is shaped not only by static traits (e.g., demographics) but also by exposure and behavioral engagement.

### Implications

First, onboarding builds *familiarity* (guided tours, practice trials with feedback) and is likely to raise appropriate trust, especially among older or less experienced users. Second, in high-stakes contexts, human-in-the-loop workflows and explicit error bounds can align trust with risk. Third, task-aware explainability tailored to memory vs. complex judgment tasks should emphasize provenance and uncertainty articulation. Finally, deployers should monitor calibration (both user and model) and publish post-deployment performance dashboards to sustain the warranted trust.

### Limitations

The cross-sectional, convenience sample limits causal inference and generalizability. Measurement invariance (e.g., multi-group CFA or DIF) was not tested; thus, some between-group differences may partially reflect scale non-equivalence rather than true trust differences. Our predictive modeling uses survey features and may not capture context-dependent shifts in trust under varying error costs or observed AI performance. Although we used nested cross-validation and calibration analyses, external validation of new populations and roles (e.g., clinicians, drivers, regulators) remains necessary.

We did not incorporate actual AI performance feedback or error-cost scenarios; future trust calibration studies should model over- vs. under-trust relative to system accuracy and risk.

## Conclusion

This study provides empirical insight into public trust in AI’s cognitive abilities across various decision-making and memory-related tasks. We identified several critical patterns by surveying 400 participants and applying a combination of statistical and machine learning methods, including MANOVA, Kruskal-Wallis H-tests, and a Random Forest classifier. The results demonstrate that AI familiarity is the most influential factor driving trust, followed by age and gender. Participants showed higher confidence in AI for tasks involving memory recall and logistics, while humans preferred high-risk domains such as healthcare and autonomous vehicles. These differences reflect a nuanced view of AI, where trust depends not only on the task but also on users’ prior experience and their perceived level of complexity.

### Future research

We propose: (i) longitudinal or randomized familiarity interventions with cross-lagged/panel models to test directionality; (ii) trust-calibration studies that incorporate ground-truth AI performance and scenario-specific error costs; (iii) multi-group CFA/DIF to establish measurement invariance across age and gender; and (iv) multi-site, cross-cultural sampling with hierarchical models to assess generalizability across stakeholder roles.

In addition, future work could leverage deep learning architectures such as convolutional neural networks for feature extraction^17^ and transformers for sequence modeling to capture nonlinear interactions among demographic, experiential, and contextual variables. These approaches can augment Random Forest classification by automatically learning richer representations from raw survey responses or interaction logs. Moreover, integrating explainable AI techniques (e.g., SHAP values, attention-based saliency maps) within these models can help maintain interpretability while enhancing predictive power. Such hybrid pipelines may enable more nuanced personalization of AI interfaces and better calibration of trust that adapts dynamically to individual user profiles and task demands.

## Data Availability

All data generated or analyzed during this study are available from the corresponding author upon request.

## References

[1] Lee, J. D. & See, K. A. Trust in automation: designing for appropriate reliance. *Hum. Factors*. **46** (1), 50–80. 10.1518/hfes.46.1.50.30392 (2004). 15151155 10.1518/hfes.46.1.50_30392

[2] Hoffman, R. R., Mueller, S. T., Klein, G. & Litman, J. Metrics for explainable AI: challenges and prospects. *Commun. ACM*. **61** (2), 68–75. 10.1145/3233231 (2018).

[3] Jussupow, E., Kehr, F. & Buchhorn, G. Explaining the unexplainable: measuring textual and visual algorithm explanations in human–AI interaction. *MIS Q.* **45** (3), 1675–1705. 10.25300/MISQ/2021/15336 (2021).

[4] Park, K. & Yoon, H. AI algorithm transparency, pipelines for trust not prisms. *Humanit. Social Sci. Commun.* **12**, 241. 10.1057/s41599-025-05116-z (2025).

[5] Dietvorst, B. J., Simmons, J. P. & Massey, C. Overcoming algorithm aversion: people will use imperfect algorithms if they can (even slightly) modify them. *Manag. Sci.* **64** (3), 1155–1170. 10.1287/mnsc.2016.2643 (2018).

[6] Afroogh, S., Zhang, J. & Wong, M. Trust in AI: progress, challenges, and future directions. *Humanit. Social Sci. Commun.* **11**, 366. 10.1057/s41599-024-04044-8 (2024).

[7] MitznerTL, Stuck, R., Hussain, S. & Rogers, W. A. Older adults’ trust in intelligent virtual agents. *ACM Trans. Hum.-Comput. Interact.* **26** (3), Article18. 10.1145/3351255 (2019).

[8] Longoni, C., Bonezzi, A. & Morewedge, C. K. Resistance to medical artificial intelligence. *J. Consum. Res.* **46** (4), 629–650. 10.1093/jcr/ucz013 (2019).

[9] Li, T., Yang, J., Zhang, Y. & Lee, D. Overconfident and unconfident AI hinder human–AI collaboration. arXiv [preprint]. (2024). https://arxiv.org/abs/2402.07632

[10] Bullock, J., Pauketat, J. V., Huang, Y., Wang, B. & Anthis, J. R. Public opinion and the rise of digital minds: perceived risk, trust, and regulation support. arXiv [preprint]. (2025). https://arxiv.org/abs/2504.21849

[11] Zhang, B. & Dafoe, A. Artificial intelligence: American attitudes and trends. *AI Soc.* **37** (4), 845–859. 10.1007/s00146-021-01306-0 (2022).

[12] Cheng, M., Nazarian, S. & Bogdan, P. There is hope after all: quantifying opinion and trustworthiness in neural networks. *Front. Artif. Intell.* **3**, 54. 10.3389/frai.2020.00054 (2020). 33733171 10.3389/frai.2020.00054 PMC7861320

[13] Shin, D. *Artificial Misinformation: Exploring human–algorithm Interaction Online* (Palgrave Macmillan, 2024). 10.1007/978-3-031-52569-8

[14] Shin, D. *Debiasing AI: Rethinking the Intersection of Innovation and Sustainability* (Routledge, 2025). 10.1201/9781003530244

[15] Alazwari, S. et al. Computer-aided diagnosis for lung cancer using waterwheel plant algorithm with deep learning. *Sci. Rep.* **14**, 20647. 10.1038/s41598-024-20647-0 (2024). 39232180 10.1038/s41598-024-71551-8 PMC11375088

[16] Vaswani, A. et al. Attention is all you need. In *Advances in Neural Information Processing Systems*. 30. https://arxiv.org/abs/1706.03762 (2017).

[17] Alzahrani, A. A. et al. Deep structured learning with vision intelligence for oral carcinoma. *Sci. Rep.* **15**, 6610. 10.1038/s41598-025-06610-0 (2025). 39994267 10.1038/s41598-025-89971-5 PMC11850820

